# Neurobiology of depression: an integrated view of key findings

**DOI:** 10.1111/j.1742-1241.2007.01602.x

**Published:** 2007-12

**Authors:** V Maletic, M Robinson, T Oakes, S Iyengar, S G Ball, J Russell

**Affiliations:** 1 School of Medicine, University of South Carolina Greer, SC, USA; 2 Eli Lilly and Company Indianapolis, IN, USA; 3 Indiana University School of Medicine Indianapolis, IN, USA

## Abstract

**Aims:**

The objectives of the present review were to summarise the key findings from the clinical literature regarding the neurobiology of major depressive disorder (MDD) and their implications for maximising treatment outcomes. Several neuroanatomical structures in the prefrontal and limbic areas of the brain are involved in affective regulation. In patients with MDD, alterations in the dynamic patterns of activity among these structures have profound implications for the pathogenesis of this illness.

**Discussion:**

The present work reviews the evidence for the progressive nature of MDD along with associated changes in neuroanatomical structure and function, especially for the hippocampus. The role of glucocorticoids, inflammatory cytokines and brain-derived growth factors are discussed as mediators of these pathological alterations. From this integrated model, the role of antidepressant therapy in restoring normative processes is examined along with additional treatment guidelines.

**Conclusion:**

Major depressive disorder is an illness with significant neurobiological consequences involving structural, functional and molecular alterations in several areas of the brain. Antidepressant pharmacotherapy is associated with restoration of the underlying physiology. Clinicians are advised to intervene with MDD using an early, comprehensive treatment approach that has remission as the goal.

**Review Criteria**

The search strategies used for this review involved literature searches of the MEDLINE and Psychinfo electronic databases. The main heading terms included major depressive disorder, neurobiology, antidepressant, hippocampus, brain-derived neurotrophic growth factor, glucocorticoids and monoamines. As part of the research strategy, each article's bibliography was reviewed for additional potential research findings relevant to these terms.

**Message for the Clinic**

Major depressive disorder (MDD) is an illness with significant neurobiological consequences involving structural, functional and molecular alterations in several areas of the brain. Antidepressant pharmacotherapy is associated with restoration of the underlying physiology. Clinicians are advised to intervene with MDD using an early, comprehensive treatment approach that has remission as the goal.

## Introduction

Major depressive disorder (MDD) remains one of the most frequently seen psychiatric illnesses in primary care settings (1). Although family and primary care physicians have greatly increased their recognition and treatment of this illness, MDD remains an unresolved treatment challenge for many physicians and patients (2). Increasing evidence has accrued in recent years regarding the impact of MDD on the structural and functional processes occurring in the brain. From the initial views that depression was caused by ‘chemical imbalance’ in the brain, this body of research has developed into a complex theory involving neuronal networks and plasticity (3). The network model has also led to a greater understanding of the mechanisms of effective treatment interventions and their role in mitigating the deleterious effects of MDD (4).

The objectives of the present review were to summarise the key findings from the clinical literature regarding the neurobiology of MDD and their implications for maximising treatment outcomes. First, the evidence that MDD is not only a chronic and recurrent illness, but also a progressive illness will be presented. Second, the impact of MDD on the primary neuroanatomical sites associated with mood regulation will be described at the structural and functional level. Third, the molecular processes that have been implicated for mediating these structural and functional changes will be explored. Fourth, the role of multiple neurotransmitter systems will be reviewed for their involvement in restoration and recovery from MDD. The last section will discuss the treatment guidelines for obtaining remission in the context of this neurobiological model.

## Major depressive disorder as a progressive illness

Epidemiological studies have consistently shown that MDD is one of the most prevalent lifetime psychiatric disorders. In the National Comorbidity Replication Survey, based on DSM-IV criteria for MDD, the lifetime prevalence rate was 16.2%, with a 12-month estimate of 6.6% (5). The presentation of MDD is heterogeneous with respect to both core and associated symptoms (6). In the Diagnostic and Statistical Manual of Mental Disorders Fourth Edition, Text Revision (7), the diagnosis of MDD requires the experience of major depressive episodes that are defined by at least five of the following symptoms for at least 2 weeks duration: loss of interest, depressed mood, appetite/weight disturbance, sleep disturbance, psychomotor change, loss of energy, worthlessness/guilt, concentration difficulties/indecisiveness and thoughts of death/suicide. Depressed mood or loss of interest must be one of the symptoms, but with the inclusion of compound criteria (e.g. worthlessness or guilt), a diagnosis of MDD can be met by various permutations, and episodes may then be further qualified by other associated features (e.g. postpartum, seasonal pattern, with melancholy or psychotic symptoms).

Even though MDD is characterised as an episodic illness, prospective studies have found that recurrence is the norm rather than the exception. For example, in a naturalistic, 15-year follow-up of a sample of 380 patients experiencing an index MDD episode, 73% experienced a recurrent episode (8), with each subsequent episode increasing the probability of further episodes (9). Similarly, in the STAR*D Project (Sequenced Treatment Alternatives to Relieve Depression) that includes 1500 patients with MDD, 74% of patients had experienced more than one episode (10). Recurrence of MDD appears to be driven in part by neurobiological vulnerabilities. In the STAR*D Project, patients who experienced multiple episodes were more likely to have a positive family history of depressive illness and an earlier age of onset of their index depressive episode compared with patients who were in their first episode (10).

**Figure 1:**
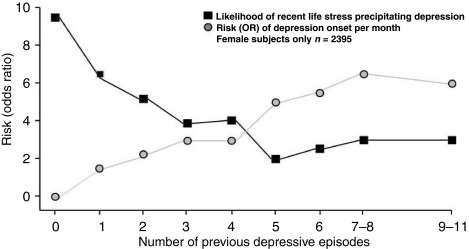
Major depression as a progressive illness. As the number of major depressive episodes increase, the risk for subsequent episodes is predicted more from the number of prior episodes and less from the occurrence of a recent life stress. Figure adapted from ref. no. (14)

Consistent evidence has also supported a ‘kindling hypothesis’ in which depressive episodes become more easily triggered over time (11). As the number of depressive episodes increase, future episodes are predicted more by the number of prior episodes rather than by life stress (12) (Figure 1). Kindling can be described as a process which occurs by a lowering of the threshold for the impact of stressful life events (i.e. sensitisation to minor events) or by an increase in spontaneous dysregulation, both of which could indicate progressive effects of MDD (13). An analysis of the risk of recurrence in a large study of twins also suggests genetic contributions as patients with a high genetic risk were ‘prekindled’; that is, they had a lower association between stressful life events and the onset of depressive episodes compared with patients having a low genetic risk (14).

Early adverse experiences may also contribute to long-term neurobiological alterations associated with depression. In preclinical studies, maternal deprivation of rat pups during critical development periods resulted in subsequent hyper-reactivity to stress and marked behavioural changes in adult rats (15). In children who had a history of early maltreatment, the risk for depressive symptoms was associated with an interaction between genotypes [e.g. serotonin (5-HT) transporter] and history of maltreatment (16). Considering these findings, some researchers have suggested that greater neurobiological changes occur in patients with depression who have early adverse experiences compared with patients who are depressed but do not have such a history, indicating that these patients may represent an especially vulnerable subtype of depressive illness (17).

Chronicity also suggests long-term neurobiological consequences associated with the MDD illness. In the STAR*D Project, 25% of the patients (with single or recurrent MDD) were identified as having a chronic episode of more than 2 years duration (10). In another large multicentre treatment study (*n* = 681), patients’ depression was classified using DSM-IV modifiers into four categories: chronic MDD (episodes > 2 years), MDD with incomplete recovery (partial response), MDD superimposed on dysthymia (double depression) and chronic MDD superimposed on dysthymia (depressive symptoms > 4 years). Despite multiple comparisons across a broad range of clinical and psychological variables, few differences were found among the four groups, resulting in the conclusion that various manifestations of chronic depression represent the same illness (18).

As the duration of depressive episodes increases, the probability of recovery substantially decreases over time. In a 5-year prospective study of outpatients with depression, approximately half recovered within the first 6 months, but afterwards the rate of recovery diminished substantially. For example, patients who had experienced depressive episodes of 1-year duration had a recovery rate of 16% compared with a 1% recovery rate for patients whose episodes persisted > 5 years (19). Similarly, in a prospective study of new onset depressive episodes, a longer duration (> 12 weeks) of previous episodes reduced the likelihood of recovery from the new onset episode by 37% (20).

Even if patients no longer meet full criteria for an MDD episode, studies have found that a substantial subset of patients continue to experience residual symptoms and diminished functioning. In a 3-year longitudinal epidemiological study, 165 patients were assessed before and after an MDD episode. Although mean values on functional measures returned to premorbid levels, 15–40% of patients experienced a worsening in psychosocial functioning that persisted after the episode, and the overall functioning of the entire sample continued to be lower than that of a healthy cohort (21). In a 10-year, naturalistic longitudinal study, patients who experienced subthreshold depressive symptoms following an MDD episode were at significantly greater risk for a recurrence, and they also had a much faster onset of their next episode compared with patients whose episode had fully remitted, suggesting that residual symptoms represent vulnerability because of an active disease state (22).

The recurrence and chronicity of MDD along with possible kindling effects have shifted the perspective of the appropriate treatment goal. The gold standard for treatment outcome has been raised from response (reduction in symptoms) to remission (absence of symptoms) or recovery (extended period of remission) (23). However, obtaining recovery implies not only the remission of symptoms but also a restoration of the underlying physiology associated with the illness. Therefore, further understanding of the neurobiological changes associated with MDD is necessary for identifying true recovery processes.

## Functional and structural changes in MDD

**Figure 2:**
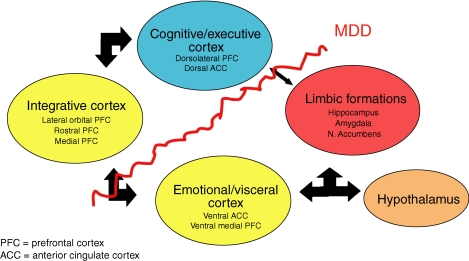
Major depressive disorder affects the dynamic connectivity among neuroanatomical structures involved in regulation of mood and stress response. Limbic structures (amygdala, hippocampus and nucleus accumbens) have reciprocal connections with ‘para-limbic’ cortical areas, subgenual anterior cingluate and ventromedial prefrontal cortex (VMPFC). Hypothetically, disrupted ‘connectivity’ between limbic/para-limbic areas and rostral integrative prefrontal formations, results in compromised feedback regulation of limbic activity. Consequently, dorsal cognitive/executive network is hypoactive while overly active limbic areas continue to stimulate the hypothalamus leading to neuroendocrine dysregulation and sympathetic hyperactivity

Although much information still needs to be attained, imaging and other methods have begun to elucidate the neurobiological abnormalities associated with MDD. In particular, several prefrontal and limbic structures and their interconnected circuits have been implicated in affective regulation (Figure 2). These neuroanatomical areas include the ventromedial prefrontal cortex (VMPFC), lateral orbital prefrontal cortex (LOPFC), dorsolateral prefrontal cortex (DLPFC), anterior cingulated cortex (ACC), ventral striatum (including nucleus accumbens), amygdala and the hippocampus. Abnormalities in these areas have been shown in patients with MDD compared with healthy controls and thus suggest a foundation for the symptomatic expression of MDD (24, 25). However, these deviations may be obscured or not present at the individual patient level and thus, these findings cannot necessarily be considered pathognomic.

As an integrated circuit, the prefrontal cortex, cingulate, amygdala, and hippocampus serves not only mood regulation, but also learning and contextual memory processes. Within the prefrontal cortex, the VMPFC mediates pain, aggression, sexual functioning and eating behaviours whereas the LOPFC assesses risk and modulates maladaptive and perseverative affective states (behaviours). These two areas have a reciprocal pattern of activity with the DLFPC, which maintains executive function, effortful sustained attention, and working memory processes (26). Subdivisions within the ACC assume diverse roles, with the dorsal ACC being part of the cognitive/executive functioning network and the ventral ACC being involved in assessing emotional and motivational information. The ACC also monitors outcomes of behaviour and cognition and makes adjustments based on changing contingencies (27, 28).

In patients with MDD, regional blood flow studies suggest hyperactivity in the VMPFC and LOPFC and hypoactivity in the DLFPC compared with controls (24). Given the functions of these regions, as previously described, this abnormal activity pattern may be responsible for the manifestations of symptoms associated with MDD. Hyperactivity of the VMPFC is associated with enhanced sensitivity to pain, anxiety, depressive ruminations and tension whereas hypoactivity of the DLFPC may produce psychomotor retardation, apathy, and deficits in attention and working memory. Using fMRI paradigms, connectivity studies have also suggested a decrement in the ‘communication’ between amygdala and ACC regions (29). A consequence of this loss of connectivity could be a failure of the ACC to serve its inhibitory role in emotional regulation (30), resulting in further motivational and affective disruption (31).

At the intersection of limbic, cognitive/executive and neuroendocrine regulatory circuits, including the hypothalamic-pituitary-adrenal axis (HPA), the hippocampus may be particularly vulnerable in depression. Imaging studies of hippocampal volume have been of particular interest. In a meta-analysis of 12 studies, hippocampal volume was found to be consistently and significantly reduced in patients with MDD compared with controls, and these reductions occurred bilaterally with a slightly greater decrement in right hippocampal volume (32). Other studies have shown that the degree of hippocampal reduction is directly proportional to the number and the duration of untreated depressive episodes (33). Among depressed inpatients, while controlling for the effect of age, hippocampal volume was significantly correlated with duration of illness prior to hospitalisation (34). Even after remission of an episode, patients with recurrent MDD have continued to show significantly smaller hippocampal volume compared with healthy controls (35).

Differences in hippocampal volume between patients with depression and healthy controls may not be fully attributable to the disease state. Heritability studies of hippocampal volume suggest both environmental and genetic contributions with heritability estimates of 54% in nonhuman primates and 40% in adult male twins (36, 37). Several genomic imaging studies, comparing patients with MDD and healthy controls, have shown associations between hippocampal volume and specific genes that are implicated in mood disorders (38, 39). In a 1-year prospective study of 30 patients with MDD, hippocampal volume did not significantly change during the study period, but patients whose depression failed to remit had a significantly smaller hippocampus at baseline and at 1 year than did patients who did remit (40). Combining the evidence from these genetic, cross-sectional, and clinical treatment studies suggests that morphological differences in the hippocampus may be a predisposing factor in MDD, but changes can also accumulate in the course of the disease and thereby create an obstacle to full recovery.

## Molecular processes mediating neurobiological changes

The alteration in the hippocampus signifies a potential outcome of injurious feedback that occurs via neuroendocrine dysregulation. A consistent finding in patients with MDD is a high level of the stress hormone cortisol, which may cause impairment in neuroplasticity and cellular resistance (41). An imbalance between glucocorticoid and mineral corticoid receptors in MDD along with high-density glucocorticoid receptors (GRs) may also contribute to the hippocampus’ susceptibility to neuronal damage (42). Subsequent hippocampal atrophy could result in further neuroendocrine dysfunction and hence a potential ‘run-away’ system (43). Postmortem comparisons of brain tissue in patients with MDD and age-matched healthy controls have shown hippocampal shrinkage in depressed subjects that was caused by increased density of neuronal cells and a significant reduction in neuropil (i.e. decreased dendridic branching and spine complexities) (44).

**Figure 3:**
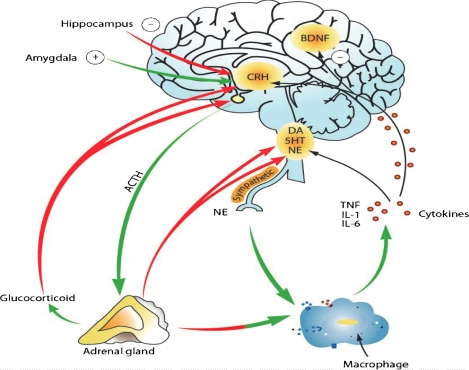
Molecular processes are impacted by stress and depression. Stress results in release of glucocorticoids and corticotrophin releasing hormones (CRH) and pro-inflammatory cytokines (TNF, IL-1, IL-6). In depression, disruption of serotonin (5-HT), norepinephrine (NE) and dopamine (DA) transmission impair the regulatory feedback loops that ‘turn off’ the stress response. Sympathetic overactivity contributes to immune activation and release of inflammatory cytokines. Inflammatory cytokines further interfere with monoaminergic and neurotrophic signalling. They may also diminish central corticosteroid receptor sensitivity, leading to disruption of feedback control. Figure adapted from ref. no. (46)

A corollary of elevated glucocorticoids and compromised hippocampal functioning may also be the down-regulation of the GR sensitivity. Under conditions of chronic stress, decrease in GR sensitivity can have negative consequences as GR signalling becomes insufficient to ‘turn off’ the initial responses to stress as part of a negative feedback process (45, 46) (Figure 3). Subsequently, HPA hypothalamic overactivity, in conjunction with amygdala activation, leads to increased sympathetic tone, which promotes the release of cytokines from macrophages. Increase in pro-inflammatory cytokines has been associated with loss of insulin and GR sensitivity, which further perpetuates metabolic and neuroendocrine disruption (47). Symptomatically, disruptions as a result of proinflammatory cytokines may be experienced as fatigue, loss of appetite and libido as well as hypersensitivity to pain (48).

Proinflammatory cytokines may also diminish neurotrophic support and monoamine neurotransmission that can lead to neuronal apoptosis and glial damage. Alterations in glia–neuron relationships have been recently emphasised in the aetiology of neuropathic pain and MDD (47, 49). Glia cells are involved in an intricate interaction with neurons in which astroglia and microglia maintain homeostasis of the neuronal environment by modulating electrolytes, neurotransmitters, cytokines and neurotrophic factors (50). Neurons reciprocate support of glial function via neurotrophin signalling. Stress, depression and ensuing peripheral immune dysregulation lead to activation of microglia that then contribute to the existing immune disruption by additional release of inflammatory cytokines (51).

An integral part of maintaining the health of these glial–neuron interactions may be mediated by brain-derived neurotrophic factor (BDNF) (52). Involved in neurogenesis, BDNF is the primary neurotrophin of the hippocampus. As a dimeric protein involved in cell maintenance, plasticity, growth and death (apoptosis), BDNF is structurally related to nerve growth factor and is distributed widely throughout the brain (53). When BDNF interacts with tyrosine receptor kinase receptors (TRkB), it promotes cellular resilience and long-term potentiation. However, the precursor form of BDNF (pro-BDNF) can also precipitate reduction in dendritic spines and cell death when it binds with the p75 receptor. Thus, depending upon its expression, BDNF can prune neural networks in an activity dependent manner that is regulated by various neurotransmitters [glutamate, GABA, 5-HT, norepinephrine (NE), acetylcholine, dopamine and hormones] (54).

Preclinical and clinical studies have suggested dysregulation in BDNF occurs under conditions of chronic stress and depression. In animal models, acute and chronic immobilisation stress resulted in decreased BDNF expression using mRNA assays. Similar results were also observed following administration of acute and chronic pain stimuli (55). Within humans, levels of serum BDNF has been found to be significantly lower in untreated patients with MDD compared with treated patients or healthy controls (56). Similarly, postmortem analyses of brains of persons who committed suicide showed that BDNF and another neurotrophin (NT-3) were significantly reduced compared with non-suicide controls (57).

From the above observations, the neurotrophic hypothesis has emerged as a major theory for the pathogenesis of major depression. In this model, stress and genetic vulnerability elevate glucocorticoid steroids and alter cellular plasticity via downregulation of growth factors and receptor sensitivity (4). The reduction in growth factors, such as BDNF, impacts negatively on the structural and functional processes within the limbic system, especially for the hippocampus. Chronic and recurrent MDD may result in subsequent atrophy and further disruptions in neurocircuitry. From this hypothesis, recovery and remission of MDD would be dependent upon a reversal of these processes, such as an increase in BDNF levels.

Complementing the neurotrophic hypothesis of MDD is the monoamine theory, which postulates that depression is associated with low levels of monoamines, particularly, 5-HT and NE. A recent imaging study of patients with untreated depression found a high global receptor density for the monoamine oxidase A (MAO-A), which nonspecifically metabolises these neurotransmitters. In this updated theory, long-term monoamine loss because of this global MAO-A activity interacts with regional specific transporter densities (i.e. 5-HT, NE), resulting in the expression of the depressive illness (58). Both 5-HT and NE ascending fibres originate from brainstem nuclei and innervate the limbic system, prefrontal cortex and associated structures involved in the regulation of mood. Descending pathways project through the dorsolateral spinal column and are instrumental in the regulation of pain (59, 60). Therefore, depending upon the specific transporter densities within these regions, various symptoms of depression (mood, cognition and pain) will be manifested within the context of the overall global reduction in monoamine levels (58).

## Role of neurotransmitters in recovery from MDD

Therapeutically, selective serotonergic reuptake inhibitors (SSRIs) and NE reuptake inhibitors (NRIs) are known to increase their respective monoamine levels in the brain. Chronic treatment with monoamine reuptake inhibitors increases activation of cyclic adenosine 3-5 monophosphatase (cAMP), which in turn stimulates protein kinase A. Activation of this protein enzyme regulates target genes leading to an increase in BDNF synthesis (52). The antidepressant-induced cAMP activity can also enhance GR sensitivity and inhibit cytokine signalling, further assisting in the restoration of the neurocircuitry feedback loops (61).

**Figure 4:**
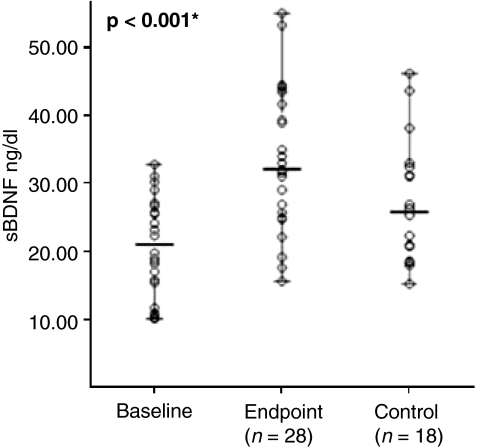
Antidepressant therapy is associated with restoring normative processes. Treatment with various selective serotonin antidepressant treatments and serotonergic noradrenergic reuptake inhibitors resulted in increases in serum brain-derived neurotrophic factor (BDNF) for patients with MDD to levels comparable that were observed with healthy controls. Reprinted with copyright permission from ref. no. (66)

The effect of increasing monoamine levels (dopamine, 5-HT and NE) on BDNF and growth factors may be one mechanism that produces the antidepressant response. Preclinical study of rat brain cells has demonstrated that monoamenergic activity (NE, 5-HT) upregulates BDNF synthesis in astrocytes (62). Clinically, successful treatment with antidepressants results in normalisation of serum BDNF level, which is considered an indirect measure of cortical BDNF activity. Support for the relationship between serum and cortical BDNF levels has been derived from correlations in animal studies as well as findings that serum BDNF passes the blood–brain barrier and reflects stored and circulating BDNF in humans (63, 64). In a study of 10 patients who were treated for 12 weeks with a dual reuptake inhibitor, improvement in depressive symptoms was correlated with increases in BDNF levels, and the BDNF levels of remitted patients had normalised to the same level observed in healthy controls (65). Response to various SSRI and 5-HT noradrenalin reuptake inhibitors (SNRI) treatments has been similarly associated with restoration of normative BDNF values (66) (Figure 4). Postmortem analysis of brain tissue has shown that subjects who had been treated with an antidepressant at time of death had greater hippocampal BDNF expression as measured by immunoreactivity than did untreated subjects with mood disorders (67).

Antidepressant therapeutic response is also associated with re-establishment of normative cortical activity. A study of 17 inpatients with MDD examined regional activity changes following 1 week and 6 week fluoxetine treatment. At 1 week, all patients showed increases in hippocampal activity and decreases in posterior cingulate and prefrontal cortex activity. After 6 weeks of treatment, patients who had responded to treatment showed a reversal of this pattern with decreased limbic activity and increased prefrontal cortical activity whereas non-responders continued to show the 1-week pattern (68). Normalisation in the amygdala and ACC has also been associated with positive response to treatment. Using a masking paradigm for subconscious activation, patients with MDD showed a baseline hyper-reactivity of the left amygdala that attenuated following 8-week treatment with sertraline (69).

Other lines of evidence also support the restorative nature of antidepressant therapy. Structural and functional MRI assessments of patients with MDD who were treated with fluoxetine indicated the importance of ACC grey matter volume for treatment response as there was a positive association among grey matter volume, normalisation of ACC activity, and response to treatment (70). Conversely, in patients with MDD who failed to respond to antidepressant treatment, plasma levels of proinflammatory cytokines were elevated compared with healthy controls or euthymic patients with MDD (71).

Symptomatically, improvements in specific MDD symptoms have been associated with regional improvements in brain metabolic activity. In 39 outpatients with MDD, improvement in cognitive symptoms was correlated with increases in DLPFC and improvements in fatigue/psychomotor retardation was associated with decreases in VMPFC activity. Interestingly, these changes were seen in responders regardless of whether treatment was pharmacological or psychological (72). Restoration of the neurobiological regulation in MDD via neurotrophic factors and neurogenesis appears to be a common factor across various effective treatments for MDD, including pharmacological, psychological and somatic treatments, such as diet and exercise (73).

## Treatment implications of the neurobiological model

The neurobiological sequelae and repercussions of chronic or recurrent MDD indicate that interventions for MDD should be focused on achieving optimal treatment early. Longitudinal studies have shown that one of the best predictors of remission status at 2 years was response to acute treatment, i.e. initial 6 weeks (74). In addition, the adequacy of treatment may also have prognostic implications. For patients with late-life depression, exposure to previous inadequate trials of antidepressants resulted in a reduced response rate to pharmacological intervention augmented by psychotherapy compared with treatment of naive patients, even after controlling for baseline severity (75). Similarly, in a large observational study of 996 patients with MDD, non-response or incomplete response to initial antidepressant treatment was a significant predictor of eventual treatment resistance (76). On the positive side, an early response to antidepressants has been shown to predict greater treatment adherence (77).

One way of maximising early response is to apply a comprehensive treatment that increases activity of multiple monoaminergic systems. In a double-blind, randomised treatment study, 39 inpatients with MDD received either fluoxetine (a serotonergic intervention), desipramine (a noradrenergic intervention) or their combination. After 6 weeks of treatment, patients who had been given the combination treatment were more likely to achieve remission (53.8%) than either intervention alone (0 % and 7.1%) (78). Similarly, a recent large meta-analysis encompassing 93 trials and 17,036 patients compared efficacy outcomes of SSRI with SNRI treatments for MDD that showed a modest but significant advantage in efficacy with SNRI treatments (79). An earlier meta-analysis did not find a difference in efficacy between SSRIs and dual acting agents (mostly tricyclic antidepressants), with the exception of the inpatient populations, where dual acting tricyclic antidepressants had an advantage (80). Thus, although current treatment algorithms for MDD usually are initiated with SSRIs, the role of combination treatment or dual reuptake inhibitors are increasingly being considered as a preferred option (81).

Another advantage of targeting both of 5-HT and NE systems is improvement not only in the core features of MDD, but also in associated physical symptoms. Painful physical symptoms are prevalent in patients with MDD, and these symptoms increase the illness burden and impair the ability to attain remission (82, 83). In a study of primary care patients with MDD who were treated with SSRIs for 9 months, mood symptoms continued to improve over time while painful physical symptoms persisted (84). The occurrence of painful physical symptoms and MDD reflects the shared underlying pathophysiology between mood and pain regulation. Importantly, there may be also a synergistic interaction between the 5-HT and NE systems to obtain analgesia. In an animal model of pain, treatment with dual reuptake inhibitors or combination treatment (5-HT/NE) appeared to enhance the effectiveness of pain alleviation (85). Clinically, patients with MDD who experienced a 50% or greater reduction in pain were more likely to achieve remission than patients whose pain reduction was < 50% (86).

With remission and recovery as the goal, the treatment guidelines derived from the neurobiological model emphasise the need for not only early and comprehensive intervention, but also vigorous attention to residual symptoms. In a 2-year study of outpatients with MDD, patients who obtained only a partial remission of symptoms were more likely to relapse (67.5%) than patients who had attained full remission (15.2%) (87). Specific recommendations for the treatment of residual symptoms have not been determined empirically, but likely require additional augmentation with other pharmacological and psychological treatments; in addition to reducing the risk of relapse, the treatment of residual symptoms may enhance compliance and long-term outcomes (88).

## Conclusions

As the underlying neurobiological model of depression is increasingly understood, treatment providers are directed to recognise that the factors that may initiate a MDD episode and those that maintain the illness are likely to be very different. Genetic and stress vulnerabilities interplay to initiate a cascade of neurobiological alterations that disrupt a dynamic system. Progressive effects of recurrent and chronic MDD may then be potentiated by further structural and functional abnormalities.

Given these long-term consequences, an essential objective of treatment must be to restore normative functioning and prevent further neurobiological structural alterations. Increasing 5-HT and NE neurotransmission is likely to initiate true recovery with the restoration of neurotrophic support, glucocorticoid signalling and neuroendocrine regulation. The use of dual reuptake inhibitors enhances the probability of remission as it addresses the complex interplay of the emotional and physical symptoms of MDD. Painful physical symptoms are increasingly recognised as having a significant impact on functioning and recovery; thus, affirming the need for antidepressant treatments that can effectively reduce these symptoms as well.

From the neurobiological model, the treatment guidelines of early, comprehensive and progressive treatment require a change in perspective for both patients and providers. A residual symptom may be interpreted as a proxy of an active disease state, with ensuing structural alterations and systemic consequences. With remission and recovery as the goal, patients will need to be educated about the benefits of long-term treatment rather than episodic or incomplete intervention. A biopsychosocial treatment model that incorporates cognitive-behavioural or interpersonal therapy along with pharmacological interventions serves to address both the initiation and maintenance factors and can reduce the risk of relapse (89). Once remission is attained, maintenance of effect may become the more appropriate term, rather than relapse prevention, to emphasise the necessity for an ongoing collaboration between patient and physician in order to maintain neurobiological homeostasis.
